# Determination of mine fault activation degree and the division of tectonic stress hazard zones

**DOI:** 10.1038/s41598-024-63352-w

**Published:** 2024-05-30

**Authors:** Tianwei Lan, Yonghao Liu, Yongnian Yuan, Ping Fang, Xiangdong Ling, Chuang Zhang, Yabin Li, Yang Li, Wei Feng

**Affiliations:** 1 College of Mining, Liaoning Technical University, Fuxin, China; 2 Ordos Institute of Liaoning Technical University, Ordos, China; 3 Inner Mongolia Limin Coal Co., Ltd., Wuhai Energy Group, CHN Energy, Ordos, China; 4 Ordos Haohua Hongqingliang Mining Co, Ltd, Beijing Haohua Energy Group, Beijing Energy Group, Ordos, China

**Keywords:** Natural hazards, Energy science and technology, Geology, Structural geology, Solid Earth sciences, Tectonics

## Abstract

This article conducts a comprehensive study on the activation characteristics of faults in the mine and analyzes the distribution patterns of the original rock stress field. Through quantitative research and analysis, we determine the partitioning characteristics of tectonic stress in the mine field under the dual effects of fault activation and original rock stress. The study also reveals the significant impact of different fault activation characteristics and different tectonic stress partitions on the stability of roadway surrounding rock. Using the Mohr–Coulomb strength criterion as a foundation, we investigate the mechanisms of fault activation and establish a mathematical model for fuzzy comprehensive evaluation. This model enables us to determine the strength level of fault activation in coal seam 9 of the Limin coal mine and construct a geological structure model. It has realized the transformation of fault activation degree from qualitative evaluation to quantitative evaluation. The stress state analysis software is used to draw the division of tectonic stress dangerous areas under the synergistic effect of fault activation and original rock stress. We then analyze the impact on the stability of roadway surrounding rock in these different hazardous areas. Utilizing the fuzzy comprehensive evaluation method, we take into account the impact of faults on the distribution characteristics of stress fields and the stability of roadway surrounding rock. This approach enables us to more accurately and comprehensively determine the hazardous areas of tectonic stress in the mine field under the dual effects of faults and original rock stress.

## Introduction

In-situ stress is the fundamental driving force affecting the stability of the roadway surrounding rock. The direction and size of in-situ stress have an important influence on the stability of the roadway surrounding rock^1^. It is of great significance to analyze the characteristics of ground stress field for roadway surrounding rock support and mine safety and efficient production. In recent years, domestic and foreign scholars have conducted thorough research on the distribution characteristics of in-situ stress^2^, and the distribution characteristics of ground stress field have become clearer and the in-situ stress has become more understood. Kang et al.^3,4^ took Jincheng mining area as the research background, carried out in-situ stress measurement and distribution characteristics research, collected 1357 mine in-situ stress test data, proposed ‘ China underground mine in-situ stress database ’, and drew the in-situ stress distribution map of China mining area. Han et al.^5^ used the stress relief method to measure the in-situ stress, and obtained the distribution characteristics of the in-situ stress field in Huainan mining area, and explained that the in-situ stress field in this mining area belongs to the quasi-static horizontal stress field type. Wu et al. ^6^ used the hydraulic fracturing method to measure the in-situ stress in the peripheral exploration area of Panji coal mine in Huainan coalfield and determined that the in-situ stress state in the exploration area was dominated by horizontal stress, and the tectonic stress increased with the increase of depth. Zhu et al. ^7^ used the hydraulic fracturing method to measure and invert the in-situ stress in the deep shaft engineering area of Sanshandao. The original stress state and distribution law of nearly 2000 m ultra-deep strata were determined by field measurement and FLAC3D numerical simulation analysis. The results show that the stress level of 2000 m depth is close to or exceeds the yield strength of rock mass after excavation disturbance, and the maximum and minimum horizontal principal stress directions in the original rock stress field are nearly horizontal, which directly affects the deep stope layout and roadway direction. Accurately obtaining the in-situ stress information in the stratum is the premise of stability analysis and support design of roadway surrounding rock^8^. At the same time, fault destabilization also has decisive influences on the roadway envelope under complex fault endowment, original rock stress environment and mining disturbance.

A fault structure is a phenomenon in which a geologic body ruptures during the tectonic movement of the earth’s crust, resulting in a loss of its continuity and integrity^9^. At the beginning of the 20th century, domestic and foreign scholars recognized the hazards of faults and started to use theoretical analysis and numerical simulation to study the activation law of faults, so as to find out the importance of the damage law of faults^10,11^. In addition, under the coupling effect of internal and external geodynamic environment^12^, there will inevitably be the danger and inevitability of mine dynamic disasters, but not all faults will occur dynamic phenomena, dynamic phenomena always occur in places conducive to development^13^. Through the analysis of topography and geomorphology, the number of faults in the study area is determined, the degree of fault activation and the movement of faults on the mine are evaluated, and the possible geological dynamic effects of engineering activities are predicted. It is of great significance to evaluate the fault activation and determine the degree of its activity for predicting and preventing the dynamic phenomenon of the mine. Zhang et al.^14^ used UDEC numerical simulation software to simulate the movement and failure process of overlying strata under the conditions of different dip angles and different horizontal fault distances of small active faults. The results show that the larger the horizontal fault distance and the larger the dip angle of the small active fault, the greater the risk of overlying strata caving when the working face passes through the fault. Yin et al.^15^ applied the fuzzy comprehensive evaluation method to simulate the mining process of the working face and carried out a comprehensive analysis of the stress in different areas to judge their impact hazards, which belongs to the “pre-mining” evaluation and realizes quantitative evaluation of the impact factors of ground pressure. The evaluation results reflect the distribution of impact hazardous areas in the working face, which helps guide the prevention and control of impact ground pressure in the working face.

Therefore, this paper adopts the fuzzy comprehensive evaluation method^16^ to evaluate the activation degree of the No. 9 coal seam fault of Limin Coal Mine, which realizes the quantitative evaluation of the activation characteristics of the mine fault. The tectonic stress danger zone under the dual action of fault activation and original rock stress is determined, and reveals the influence of different fault activation characteristics and different tectonic stress zones on the stability of the tunnel enclosure. The evaluation results can well reflect the dangerous area of tectonic stress in the mine field, which is basically consistent with the actual situation on site and is conducive to guiding the safe and efficient production of the mine.

## Influence mechanism of fault activation

### Mechanism of fault activation discrimination

The results show that when the rock undergoes destabilizing damage, the angle of internal friction (*φ*) does not change significantly, while the cohesion (*c*) decreases substantially. When the fault is in limiting equilibrium, the positive stress (*σ*) and shear stress (*τ*) at the fault surface satisfy the Mohr–Coulomb strength criterion^17^.

1 $$\tau = c + k\sigma ,(\;k = {\text{tan}}\varphi )$$

In the formula: c-fault plane cohesion, MPa; k-fault friction coefficient.

When the internal friction (*φ*) angle of the fault changes little, the slope of the straight line of the fault surface remains unchanged. Under the mining disturbance, the cohesion (*c*) of the fault surface gradually decreases and the straight line decreases as a whole. There is no difference between the stress circle of each point in the fault surface and the center (*O*_1_) and radius (*R*) of the Mohr–Coulomb stress circle, as shown in formula (2), where *σ*_1_ is the maximum principal stress, *σ*_3_ is the minimum principal stress, and Pi is the fluid pressure of the fault fracture.

2 $$O_{1} = \left( {\frac{{\sigma_{1} + \sigma_{3} - 2{\text{p}}_{i} }}{2},0} \right),\;R = \frac{{\sigma_{1} - \sigma_{3} }}{2}$$

**Figure 1:**
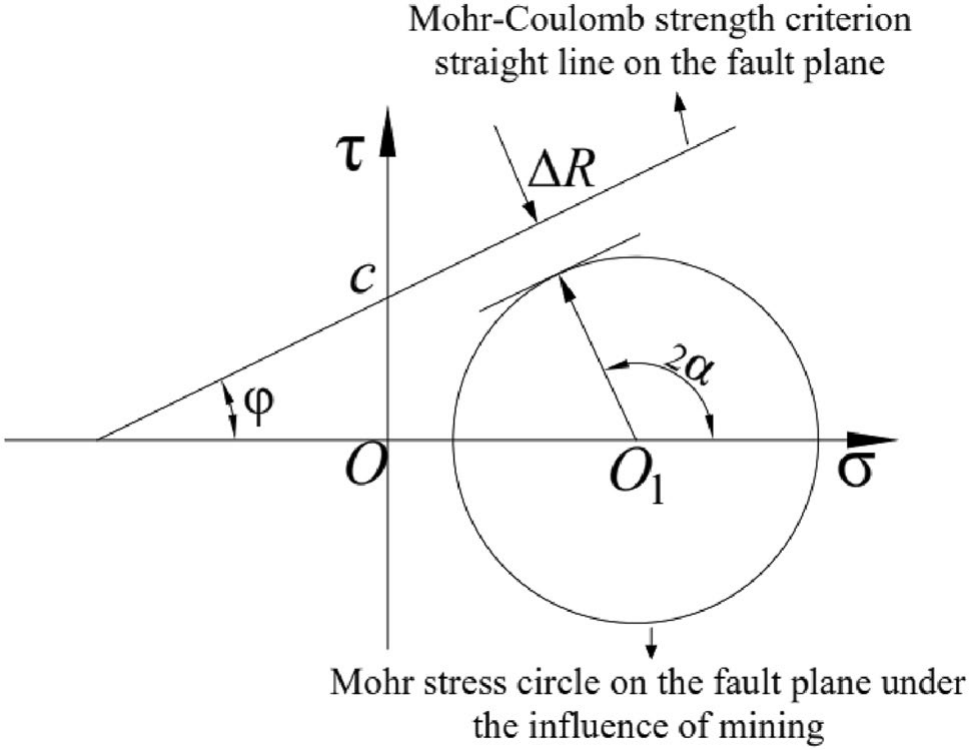
Schematic diagram of fault activation discrimination.

The discrimination of fault activation is illustrated in Fig. 1, $\overline{{OO_{1} }} = \left( {\sigma_{1} + \sigma_{3} - 2{\text{p}}_{i} } \right)/2$. Assuming the Mohr stress circle on the fault plane under the influence of mining activity intersects with the straight line representing the Mohr–Coulomb strength criterion, the vertical distance between them, denoted as Δ*R*, plays a crucial role in assessing fault activation. This distance is expressed mathematically in formula (3), offering a precise and quantitative measure for comparing and contrasting fault activation states.

3 $$\Delta R = c\cos \varphi + {\text{sin}}\varphi \frac{{\sigma_{1} + \sigma_{3} - 2{\text{p}}_{i} }}{2} - \frac{{\sigma_{1} - \sigma_{3} }}{2}$$

The influence of mining disturbance leads to continuous changes in in-situ stress magnitude. As a result, the straight line represented by the Mohr–Coulomb strength criterion on the fault plane decreases overall. Additionally, the center and radius of the Mohr stress circle on the fault plane also undergo dynamic changes. There can be three states between the Mohr stress circle on the fault plane and the straight line represented by the Mohr–Coulomb strength criterion on the fault plane, corresponding to the three states of fault activation^18^.

1. When Δ*R* > 0, the Mohr stress circle on the fault plane deviates from the straight line represented by the Mohr–Coulomb strength criterion on the fault plane, indicating that the fault is in a stable equilibrium state.
2. When Δ*R* = 0, the Mohr stress circle on the fault plane intersects the straight line represented by the Mohr–Coulomb strength criterion on the fault plane, indicating that the fault is in the limit equilibrium state.
3. When Δ*R* < 0, the Mohr stress circle on the fault plane intersects with the straight line represented by the Mohr–Coulomb strength criterion on the fault plane, indicating that the fault is in an activated state.

Based on the measurement method of hollow core inclusion in Limin Coal Mine, the maximum principal stress *σ*_1_ = 20.6 MPa and the minimum principal stress *σ*_3_ = 4.55 MPa were determined in No. 9 coal seam of Limin Coal Mine. The parameters of No.9 coal seam were determined by physical and mechanical parameters, as shown in Table 1.

**Table 1:** No. 9 coal seam fault lithology classification and parameters.

Fault lithology classification	Cohesion *c*/MPa	Internal friction *φ*/°
Mudstone	5.47	46
Sandy mudstone	6.84	34
Siltstone	14.5	37

Taking DF3 fault as an example, the fault state is described in detail. DF3 fault index *c* = 14.5 MPa, *φ* = 37°. *P*_*i*_ = 10.45 MPa determined by a point of stress state^19^. Expression of it:

4 $$\Delta R = 14.5 \times \cos 37^\circ + {\text{sin37}}^\circ \times \frac{20.6 + 4.55 - 2 \times 10.45}{2} - \frac{20.6 - 4.55}{2} = 4.83$$

Δ*R* > 0, The DF3 fault is identified as being in a stable equilibrium state, and this criterion is subsequently applied to other faults for evaluation. The activation status of the fault is summarized in Table 2.

**Table 2:** Fault activation status assessment for coal seam No. 9 in Limin coal mine.

Name of fault	Cohesion/*c*	Internal friction/*φ*	Fluid pressure of the fault fracture/P_*i*_	Fault state
DF1	14.5	37	22.68	Activated state
DF3	14.5	37	10.45	Stable equilibrium state
SF26	5.47	46	20.22	Activated state
SF42	14.5	37	21.14	Activated state
DF2	14.5	37	8.17	Stable equilibrium state
DF12	14.5	37	7.95	Stable equilibrium state
DF6	5.47	46	7.52	Activated state
DF15	14.5	37	19.33	Activated state
SF4	14.5	37	18.48	Limit equilibrium state
SF36	5.47	46	6.64	Stable equilibrium state
DF7	14.5	37	18.48	Limit equilibrium state
DF17	6.84	34	10.21	Activated state
SF28	5.47	46	7.24	Activated state
SF27	5.47	46	9.13	Activated state
SF13	6.84	34	9.67	Activated state
SF10	6.84	34	8.77	Activated state

### Mechanical action mechanism of fault activation

Under the influence of mining disturbance on the working face, the shear displacement of the upper and lower walls of the fault is referred to as fault activation. This process occurs when the fault plane undergoes shear deformation due to the action of mine pressure. As a result, new fractures may develop at one or both ends of the fault, leading to its expansion^20^.

**Figure 2:**
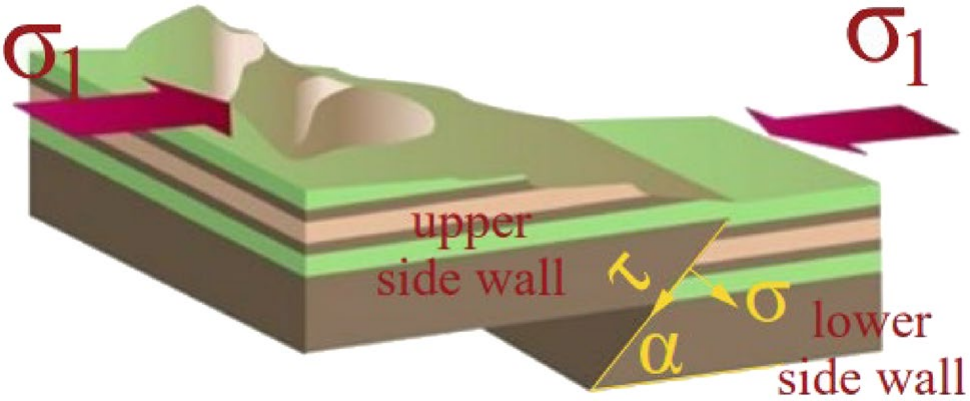
Mechanical model of fault activation.

There are two primary states of fault activation. In the first state, the cement within the fault plane is “sheared” through activation, transitioning the hanging wall and foot-wall from a “bonded” state to a “broken” state, establishing the foundation for fault activation. In the second state, the fault’s terminal and derived fractures expand through activation, significantly enhancing the permeability of the fault zone and adjacent rock masses^21^. Assuming that the upper and lower plates of the fault are composed of elastic rock masses^22^, and the fault plane serves as the contact interface between them, the initial activation of the fault is indicated by the occurrence of shear motion within the plates. A mechanical model of fault activation, as depicted in Fig. 2, is established to describe this process. The criterion for fault activation is defined as: τ ≥ τ_max_. τ_max_ is the maximum shear strength acting on the fault plane.

## Fuzzy comprehensive evaluation model for fault activity

Fuzzy comprehensive evaluation is a powerful method that utilizes the principles of fuzzy mathematics to integrate and assess multiple factors pertaining to a specific object. This approach effectively converts subjective evaluation into objective quantification, enabling a deeper and more comprehensive understanding of the object. The process involves a comprehensive evaluation of various characteristics and aspects of the object, considering all relevant factors. Obtain the final evaluation result and analyze it, providing a more accurate, objective, and comprehensive evaluation of the object. Generally, it mainly includes the following steps^23^.

1. Establish a comprehensive set of fuzzy evaluation factors.
  5 $$U = \left\{ {U_{1} ,U_{2} , \ldots U_{m} } \right\}$$
  In this process, *U*_m_ denotes the mth evaluation factor, and m represents the total number of evaluation factors in the fuzzy comprehensive evaluation.
2. Construct a fuzzy evaluation set, which specifies the evaluation grades for the fault.
  6 $$V = \left\{ {V_{1} ,V_{2} , \ldots V_{n} } \right\}$$
  Within this context, *V*_n_ denotes the nth evaluation grade, and n represents the count of evaluation grades.
3. Create a single-element evaluation matrix *A*.
  7 $$A_{i} = \left\{ {a_{i1} ,a_{i2} , \ldots a_{ij} } \right\}$$
  In this matrix, 0 ≤ *A*_ij_ ≤ 1, 1 ≤ i ≤ m, 1 ≤ j ≤ n, where *A*_ij_ represents the membership degree of the ith evaluation factor in the jth evaluation grade. The matrix *A* is the overall evaluation matrix.
  8 $$A = A_{m \times n} { = }\left| {\begin{array}{*{20}c} {\begin{array}{*{20}c} {a_{11} } & {a_{12} } \\ \end{array} } & \ldots & {a_{1n} } \\ {\begin{array}{*{20}c} {\begin{array}{*{20}c} {a_{21} } \\ \vdots \\ \end{array} } & {\begin{array}{*{20}c} {a_{22} } \\ \vdots \\ \end{array} } \\ \end{array} } & {\begin{array}{*{20}c} \ldots \\ \ddots \\ \end{array} } & {\begin{array}{*{20}c} {a_{2n} } \\ \vdots \\ \end{array} } \\ {\begin{array}{*{20}c} {a_{m1} } & {a_{m2} } \\ \end{array} } & \ldots & {a_{mn} } \\ \end{array} } \right|$$
4. Fuzzy comprehensive evaluation.
  The weight vector is employed to prioritize the allocation of various influencing factors within the set *U*. Here, the weight vector $X = \left( {x_{1} ,x_{2} , \ldots ,x_{m} } \right)$ represents a fuzzy subset of U, fulfilling the condition ∑w_*i*_ ≠ 1.

With the knowledge of the weight vector *X* and the evaluation matrix *A*, the comprehensive evaluation matrix *W* can be obtained by calculating the product of *X* and *A*, denoted as *W* = *X·A*, where *W* = 1. If ∑w_*i*_ ≠ 1, it is necessary to normalize the comprehensive evaluation matrix *W* to ensure that its sum equals 1.

Using the maximum membership degree criterion and the analytic hierarchy process, we will establish a single-element evaluation matrix *A* and a weight vector *X* to accurately classify the activation levels of various fault layers.

### Develop evaluation metrics for individual factors influencing fault activity

The evaluation indicators are crucial in ensuring the accuracy of evaluation results and essentially reflect the degree of fault activation. In line with the theory of geodynamic zoning, five specific indicators are selected to effectively determine the level of fault activation.

1. The fault drop height (*H*). The degree of fault displacement serve as indicators of fault activity. Large displacement amplitudes in the minefield indicate a highly active fault. Conversely, smaller drop heights indicate a less active fault. Therefore, it is crucial to consider the drop height of the mutual sliding on both sides of the fault when evaluating its activity level.
2. Fault dip angle (*α*). The fault dip angle exhibits a positive correlation with its activation level. As the fault dip angle decreases, the likelihood of fault instability due to sliding decreases, Conversely, as the fault dip angle increases, resulting in higher peak stress and the fault becomes more susceptible to significant dislocation, which can destabilize the surrounding rock system and enhance the risk of activation.
3. Angle between fault and maximum principal stress ($\overline{\sigma }$). The study in^24^ suggests that faults with a wavy surface can produce tectonic stress. As a result, the value of $\overline{\sigma }$ is larger for faults in the crushed zone, while it is relatively smaller for faults in the non-crushed zone and those associated with the crushed zone.
4. Angle between fault and maximum shear stress ($\overline{\tau }$). The fault activity along the fault plane can be characterized by the maximum shear stress (τ_max_). Obviously, the fault experiencing the maximum shear stress is the most active. As the angle between the fault and maximum shear stress increases, the fault activity weakens.
5. Compressive strength of fault lithology (*R*_c_). As the strength of the surrounding rock increases, the sound of failure becomes louder, indicating a higher level of fault activation. This accumulation of energy during fault activation also intensifies the released energy, elevating the risk of potential dynamic disasters.

Taking into account the significance of various factors related to fault activation, using the general criteria and methods of membership functions, individual factor-based evaluation indicators for fault activation were established. The classification ranges and average values for each factor are presented in Table 3.

**Table 3:** Individual factor evaluation indices for fault activity.

Activation classification	*H* (m)		*α* (°)		$\overline{\user2{\sigma }}$ (°)		$\overline{\user2{\tau }}$ (°)		*R*_c_ (MPa)	
	Range	Average value	Range	Average value	Range	Average value	Range	Average value	Range	Average value
High	> 50	130	60–90	75	60–90	75	60–90	75	> 10	18.4
Middle	20–50	35	30–60	45	30–60	45	30–60	45	5–10	7.5
Lower	0–20	10	0–30	15	0–30	15	0–30	15	0–5	2.5

### Developing a membership function for fault activation

Having identified the individual factor evaluation indices, it is essential to consider the specific characteristics and real-world circumstances of each index. Carefully selecting suitable techniques for determining the membership degrees of each index across various categories will facilitate the creation of a comprehensive membership function for evaluating fault activation.

Given the ambiguity in grading individual factor evaluation indices and the statistical analysis of each factor, it has been found that the probability distribution follows a normal distribution model. Therefore, this article proposes the use of a fuzzy normal distribution function as the membership function for evaluating fault activation within the same grade^25^. Expression of it:

9 $$\mu_{n} (x) = e^{{ - \left( {\frac{x - a}{b}} \right)^{2} }} (\;n =1,2,3)$$

In the provided expression, a signifies the mean value within the range of each individual factor parameter, aligning with the average values specified in Table 3 for each respective grade. Furthermore, the boundary values outlined in Table 3 for various grades serve as transitional points between adjacent grades, embodying a notion of ambiguity. Since these values inherently belong to both neighboring grades, their membership degrees are equivalent, leading us to approximate them as 0.5. Consequently, within a designated grade range, the expression for the b value is:

10 $$\frac{{X_{b} - a}}{{ \pm \sqrt {\ln 0.5} }} \approx b\;\left( {b > 0} \right)$$

In which, *X*_b_ denotes the boundary value of a specific evaluation index within a designated grade range.

Within a specific grade range, as the single-factor evaluation index has no upper limit, it follows the membership rule that when the lower limit is exceeded, the membership degree increases. Therefore, this membership function is employed for computational purposes.

11 $$\mu_{1} (x) = \left\{ {\begin{array}{*{20}l} {e^{{ - \left( {\frac{x - a}{b}} \right)^{2} }} } \hfill & {\left( {x < a} \right)} \hfill \\ 1 \hfill & {\left( {x > a} \right)} \hfill \\ \end{array} } \right.\;({\text{No}}\;{\text{previous}}\;{\text{session)}}$$

After calculation, the parameters of each membership function are presented in Table 4, specifying the final expression for determining the membership function.

**Table 4:** Parameters of each subordinate function.

Grade	Index									
	*H* (m)		α (°)		$\overline{\user2{\sigma }}$ (°)		$\overline{\user2{\tau }}$ (°)		*R*_c_ (MPa)	
	*ɑ*	*b*	*ɑ*	*b*	*ɑ*	*b*	*ɑ*	*b*	*ɑ*	*b*
High	130	96	75	18	75	18	15	18	18.4	10.1
Middle	35	18	45	18	45	18	45	18	7.5	3
Lower	10	12	15	18	15	18	75	18	2.5	3

### The AHP-entropy weight method is coupled to determine the dynamic weight coefficient

#### Weight determination by analytic hierarchy process

The hierarchical analysis method is employed to clarify fuzzy concepts, enabling the determination of evaluation indicator weights and the establishment of a fault activation evaluation weight model. M evaluation indicators are organized into a *m* × *m* matrix, and the indicators are compared pair-wise. The values of each element in the matrix are established based on the relative influence of the evaluation indicators. The maximum eigenvalue λ_max_ and the corresponding maximum eigenvector *X*_max_ are then determined. If the eigenvector passes the consistency test, it is accepted as the weight vector.

A comparison matrix was created, as presented in Table 5, based on the relative significance of each factor in assessing fault activation.

**Table 5:** Comparison matrix for individual factors.

	*H*	*α*	$\overline{\user2{\sigma }}$	$\overline{\user2{\tau }}$	*R*_*c*_
*H*	1	1.5	1.2	1.2	3
*α*	0.67	1	1.25	1.25	3
$\overline{\sigma }$	0.83	0.8	1	1	1.5
$\overline{\tau }$	0.83	0.8	1	1	3
*R*_*c*_	0.33	0.33	0.67	0.33	1

After calculations, the maximum eigenvector *X*_max_ of the matrix is obtained as (0.5818, 0.5027, 0.4019, 0.4587, 0.1918), and the maximum eigenvalue λ_max_ = 5.06.

The consistency of the comparison matrix is checked using the following expression:

12 $$CR = \frac{CI}{{RI}}$$

In this expression, *CR* serves as the random consistency index for the matrix. When *CR* < 0.1, it satisfies the criterion for consistency. *CI*, the general consistency index, is computed as *CI* = (λ_max_ − m)/(m − 1). *RI*, the average random consistency index, is obtained from Table 6.

**Table 6:** RI value.

Order number	1	2	3	4	5	6	7	8	9	10
*RI*	0	0	0.58	0.90	1.12	1.24	1.32	1.41	1.45	1.49

Calculating CI = (λ_max_ − m)/(m − 1) = (5.06 − 5)/(5 − 1) = 0.015; *RI* is obtained from Table 6, taking 1.12. *CR* = *CI*/*RI* = 0.015/1.12 < 0.1, satisfying the consistency requirement. The maximum eigenvector of the matrix is normalized and can serve as the weight vector, resulting in the weight vector *X* = (0.26, 0.23, 0.20, 0.21, 0.10).

The AHP method is used to calculate the weight of the evaluation index of the degree of fault activation, as shown in Table 7.

**Table 7:** AHP fault activation degree evaluation index weight assignment.

Evaluation index	The fault drop height (*H*)	Fault dip angle (*α*)	Angle between fault and maximum principal stress ($\overline{\user2{\sigma }}$)	Angle between fault and maximum shear stress ($\overline{\user2{\tau }}$)	Compressive strength of fault lithology (*R*_c_)
Weight	0.26	0.23	0.20	0.21	0.10

#### Entropy weight method to determine the weight

The entropy weight is to use the entropy value to calculate the degree of variation of the index, and give the weight according to the degree of variation of each index. The greater the difference between the factors, the greater the entropy weight, and the greater the impact on the evaluation results.

According to the influence of each evaluation index on the degree of fault activation, there are a total of 10 evaluation objects and 5 evaluation indexes. According to Table 10, the normalized processing formula is used to standardize the matrix *R*, and the standard *R* matrix is obtained:

13 $$R = \left[ {\begin{array}{*{20}c} {\begin{array}{*{20}c} {\begin{array}{*{20}c} {\begin{array}{*{20}c} {0.32} \\ {\begin{array}{*{20}c} \vdots \\ {0.08} \\ \end{array} } \\ \end{array} } & {\begin{array}{*{20}c} {0.78} \\ \vdots \\ {0.75} \\ \end{array} } \\ \end{array} } & {\begin{array}{*{20}c} 0 \\ \vdots \\ {0.12} \\ \end{array} } \\ \end{array} } & {\begin{array}{*{20}c} 0 \\ \vdots \\ {0.10} \\ \end{array} } & {\begin{array}{*{20}c} {0.23} \\ \vdots \\ {0.16} \\ \end{array} } \\ \end{array} } \right]$$

The information entropy *e*_*j*_ of the *j* th evaluation index is calculated as:

14 $$ej = - \frac{1}{{\ln {\text{m}}}}\sum\limits_{i = 1}^{{\text{m}}} {Pij\ln Pij}$$

In the formula, *P*_*ij*_ is the weight of the *i*-th evaluation index value under the *j-*th index.

15 $$Pij = \frac{Xij}{{\sum\nolimits_{i = 1}^{{\text{m}}} {Xij} }}$$

According to the calculation formula of the entropy weight method, the entropy value vector ej of each index is obtained as follows:

16 $$ej = \left[ {0.80,\;0.99,\;0.92,\;0.07,\;0.11} \right]$$

Calculate the difference coefficient* b*_*j*_ of the *j*-th evaluation index.

17 $$bj = 1 - ej$$

There are m evaluation objects, n evaluation indexes, and the entropy weight of the j-th index is:

18 $$W^{\prime \prime } j = \frac{1 - bi}{{\sum\nolimits_{i = 1}^{m} {bi} }}$$

Calculated by the entropy weight method, the weight vector *W*_*j*_ is:

19 $$Wj = \left[ {0.42,\;0.03,\;0.18,\;0.15,\;0.23} \right]$$

The entropy weight method is used to calculate the weight of the evaluation index of the degree of fault activation, as shown in Table 8.

**Table 8:** Entropy weight method fault activation degree evaluation index weight assignment.

Evaluation index	The fault drop height (*H*)	Fault dip angle (*α*)	Angle between fault and maximum principal stress ($\overline{\user2{\sigma }}$)	Angle between fault and maximum shear stress ($\overline{\user2{\tau }}$)	Compressive strength of fault lithology (*R*_c_)
Weight	0.42	0.03	0.18	0.15	0.23

#### The AHP-entropy weight method is coupled to determine the weight

The AHP method is essentially a subjective weighting method. Because the subjective weighting method is susceptible to the influence of expert experience and knowledge, its subjectivity is strong. In specific applications, due to the particularity, complexity and variability of objective factors, it will affect the accurate judgment of their relative importance. The entropy weight method is an objective weighting method, which overcomes human factors, but overemphasizes the internal changes between the evaluation index data, and lacks targeted analysis of the actual situation. In order to avoid the shortage of single method to calculate the index weight, the two methods are combined, and the subjective weight and objective weight of each main control factor are determined by AHP method and entropy weight method respectively. The weight results obtained by AHP method and entropy weight method are comprehensively analyzed by the combination weighting method, and the weight value more in line with the actual situation is finally obtained.

According to Formula (20), the calculation weights of the AHP method and entropy weight method are coupled, and the coupling results are shown in Table 9. The coupling weight combines the advantages of AHP method and entropy weight method, and the coupling weight highlights the decisive role of fault drop in the evaluation and prediction of fault activation degree.

**Table 9:** Different methods of fault activation degree evaluation index weight assignment results.

Evaluation index	The fault drop height (*H*)	Fault dip angle (*α*)	Angle between fault and maximum principal stress ($\overline{\user2{\sigma }}$)	Angle between fault and maximum shear stress ($\overline{\user2{\tau }}$)	Compressive strength of fault lithology (*R*_c_)
AHP	0.26	0.23	0.20	0.21	0.10
Entropy weight method	0.42	0.03	0.18	0.15	0.23
The coupling results of the two	0.53	0.03	0.17	0.15	0.11

The weights of AHP method and entropy weight method are coupled by multiplier normalization method^26^.

20 $$Wj = \frac{{W_{j}^{\prime } W_{j}^{\prime \prime } }}{{\sum\nolimits_{j = 1}^{n} {W_{j}^{\prime } W_{j}^{\prime \prime } } }}$$

### Fuzzy matrix comprehensive evaluation

Using the aforementioned method, we conducted a comprehensive evaluation of fault activity in the Limin coal mine. The fault was identified based on detailed three-dimensional geologica and in-depth supplementary survey data. Five key evaluation indicators, *H*, α, $\overline{\sigma }$, $\overline{\tau }$ and *R*_C_, were extracted and are presented in Table 10.

**Table 10:** Limin coal mine’s evaluation indicators for faults in coal seam 9.

Name of fault	Evaluation indicators				
	*H* (m)	α (°)	$\overline{\user2{\sigma }}$ (°)	$\overline{\user2{\tau }}$ (°)	*R*_c_ (MPa)
DF1	48	62	14	77	6.10
SF26	60	35	61	44	4.98
SF42	200	35	68	31	4.98
DF6	15	60	55	45	4.98
DF15	14	61	73	45	4.98
DF17	13	61	68	31	4.98
SF28	20	65	15	66	14.68
SF27	40	45	61	12	22.31
SF13	15	65	41	24	4.98
SF10	20	60	22	69	4.98

Taking the DF1 fault as an example, we calculate the membership degree of the evaluation index H = 48 m, α = 62°, $\overline{\sigma }$ = 14°, $\overline{\tau }$ = 77°, *R*_c_ = 4.98 MPa. Firstly, we substitute the membership function of H into the formula (11) of the factor set. The expression of the membership function is:

21 $$\mu_{1} (H) = \left\{ {\begin{array}{*{20}l} {e^{{ - \left( {\frac{H - 130}{{96}}} \right)^{2} }} } \hfill & {\left( {x < 130} \right)} \hfill \\ 1 \hfill & {\left( {x > 130} \right)} \hfill \\ \end{array} } \right.$$

22 $$\mu_{2} (H) = e^{{ - \left( {\frac{H - 35}{{18}}} \right)^{2} }}$$

23 $$\mu_{3} (H) = e^{{ - \left( {\frac{H - 10}{{12}}} \right)^{2} }}$$

When *H* = 48 is inserted into the membership function, the single-factor evaluation matrix *A*_H_ for the evaluation index *H* is calculated, resulting in *A*_H_ = (0.482, 0.594, 0). Using a similar approach, we can compute the membership degrees and single-factor evaluation matrices for the remaining four evaluation indicators of the DF1 fault, establishing a comprehensive evaluation matrix.

24 $$A_{5 \times 3} = \left| \begin{gathered} \begin{array}{*{20}c} {0.482} & {0.594} & {0.000} \\ \end{array} \\ \begin{array}{*{20}l} {0.594} \hfill & {0.410} \hfill & {0.001} \hfill \\ \end{array} \\ \begin{array}{*{20}c} {0.000} & {0.052} & {0.997} \\ \end{array} \\ \begin{array}{*{20}c} {0.000} & {0.042} & {0.988} \\ \end{array} \\ \begin{array}{*{20}c} {0.222} & {0.781} & {0.254} \\ \end{array} \\ \end{gathered} \right|$$

25 $$\begin{aligned} W = & X \cdot A_{5 \times 3} \\ = \; & (0.53,0.03,0.17,0.15,0.11) \cdot \left[ {\begin{array}{*{20}c} {\begin{array}{*{20}c} {\begin{array}{*{20}c} {0.482} & {0.594} & {0.000} \\ \end{array} } \\ {\begin{array}{*{20}c} {0.594} & {0.410} & {0.001} \\ \end{array} } \\ \end{array} } \\ {\begin{array}{*{20}c} {0.000} & {0.052} & {0.997} \\ \end{array} } \\ {\begin{array}{*{20}c} {\begin{array}{*{20}c} {0.000} & {0.042} & {0.988} \\ \end{array} } \\ {\begin{array}{*{20}c} {0.222} & {0.781} & {0.254} \\ \end{array} } \\ \end{array} } \\ \end{array} } \right] \\ = & \;\;(0.298,0.429,0.346) \\ \end{aligned}$$

After conducting the normalization process, we derived *W* = (0.298, 0.429, 0.346), indicating that the DF1 fault belongs to the category of weakly activated faults. Applying this method to all faults, we have determined their respective activation levels, which are presented in Table 11.

**Table 11:** Limin coal mine’s comprehensive evaluation results of fault activity in No. 9 coal mine.

Name of fault	Fuzzy comprehensive evaluation	Normalization processing	Degree of activity
DF1	W = (0.298, 0.429, 0.346)	W = (0.278, 0.400,0.322)	Middle
SF26	W = (0.435, 0.378, 0.072)	W = (0.492, 0.427, 0.081)	High
SF42	W = (0.763, 0.190, 0.065)	W = (0.750, 0.187, 0.064)	High
DF6	W = (0.219, 0.498, 0.512)	W = (0.178, 0.405, 0.417)	Lower
DF15	W = (0.335,0.369,0.439)	W = (0.293,0.323,0.384)	Lower
DF17	W = (0.369,0.302,0.554)	W = (0.301,0.247,0.452)	Lower
SF28	W = (0.261,0.322,0.551)	W = (0.230,0.284,0.486)	Lower
SF27	W = (0.565,0.502,0.003)	W = (0.528,0.469,0.003)	High
SF13	W = (0.290,0.416,0.522)	W = (0.236,0.339,0.425)	Lower
SF10	W = (0.176,0.392,0.600)	W = (0.151,0.36,0.514)	Lower

**Figure 3:**
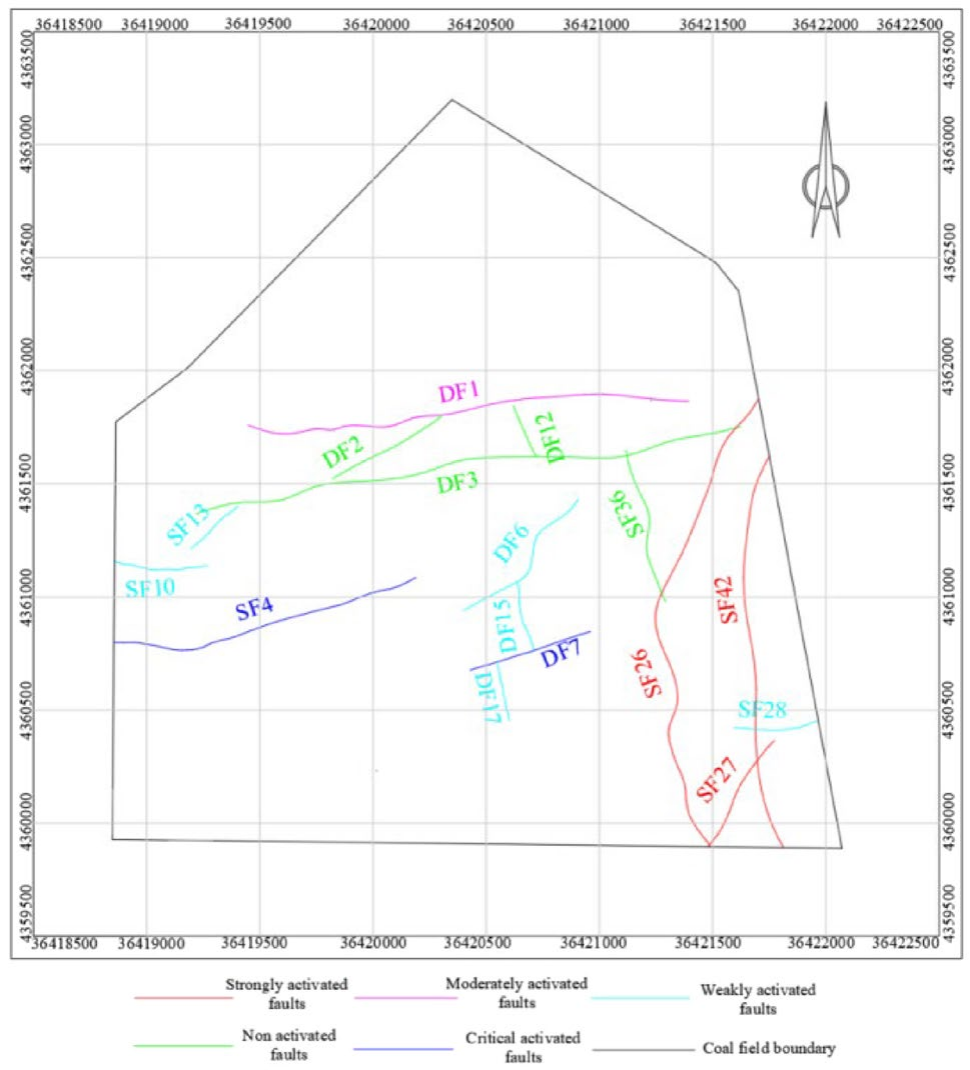
Limin coal mine’s geological structure model of No. 9 coal seam.

Using the fuzzy comprehensive evaluation method, the activation levels of all faults were determined. Based on these results, a geological structure model was established, which includes five distinct categories of active faults: non-active, critical, strongly active, moderately active, and weakly active. This model is depicted in Fig. 3.

## Analysis of tectonic stress field characteristics under the cooperative control of fault activation and original rock stress field

### Rock stress state analytical system

The geological zoning method is utilized to determine the fault structure of the minefield scale and establish the link between fault occurrences and their engineering implications. Using various fault characteristics, we have developed a comprehensive geological structure model for the No. 9 coal seam in Limin coal mine, which is depicted in Fig. 3. Based on this model, faults such as DF1, SF26, and SF42 are located within the minefield and significantly influence the stress distribution within the minefield.

**Figure 4:**
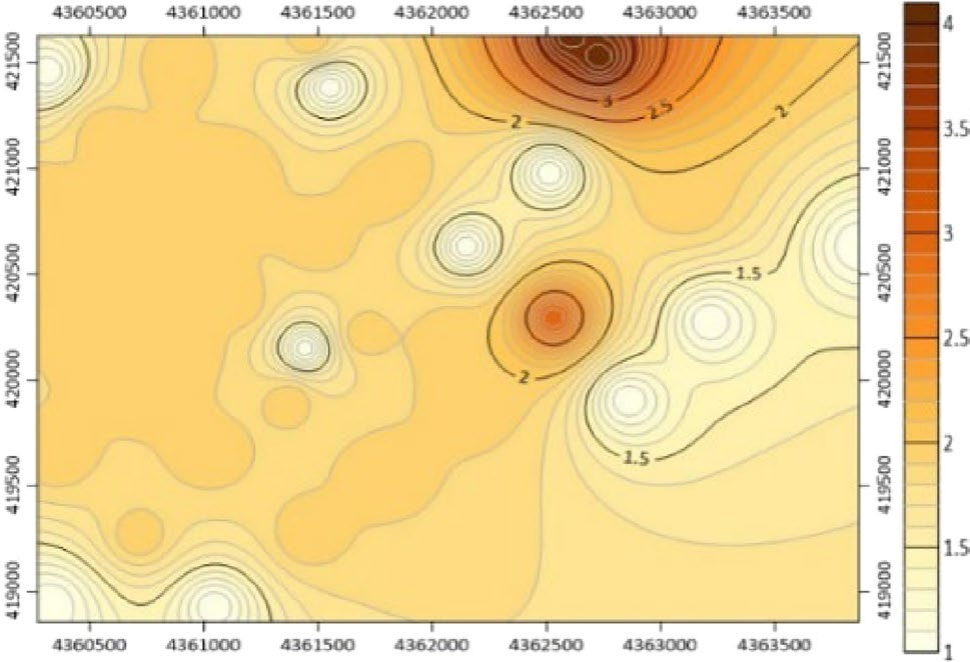
Lithology contour map of the top rock of 9 coal seam in Limin coal mine.

**Figure 5:**
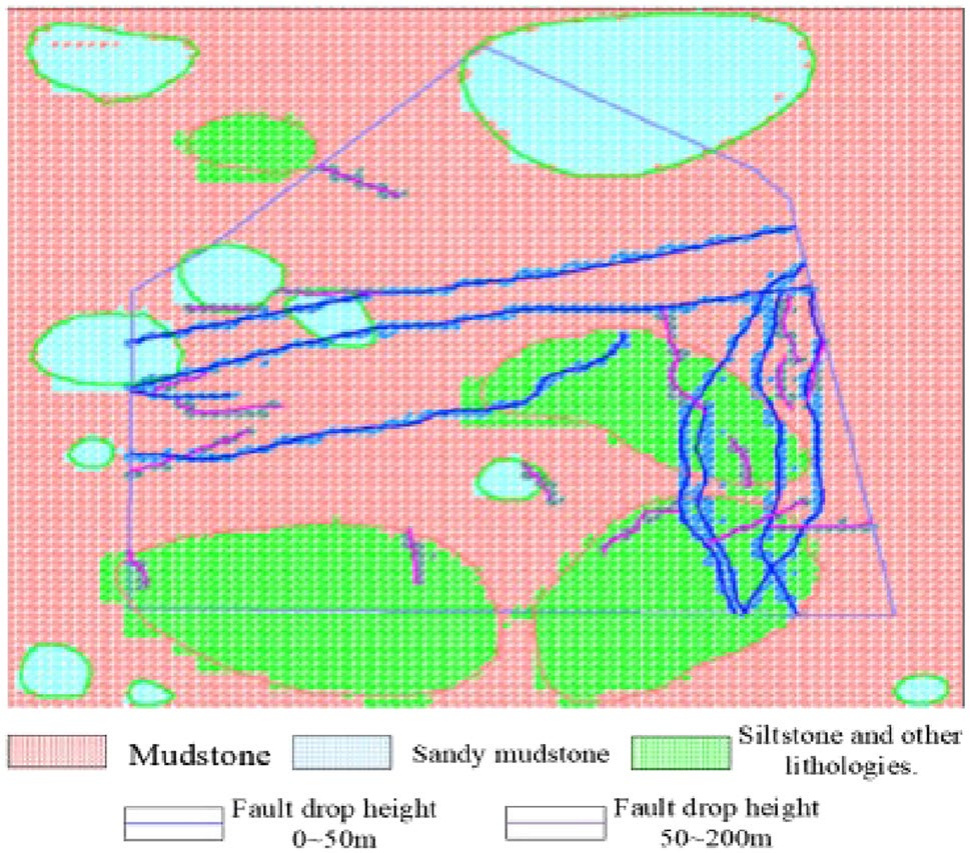
Stress calculation model and lithology distribution.

Using the rock properties data from the exploration drilling and mining exposure in the No. 9 coal seam of Limin Coal Mine, the top rock properties of the coal seam were meticulously classified and a comprehensive top rock properties code database was established, as depicted in Fig. 4. Utilizing our in-house developed CAD drawing software with a built-in plugin LP, we obtained geological structures, rock properties distribution, and mine boundary data, which were then processed into interactive visual files. These visual files were imported into our independently developed “Rock Mass Stress State Analysis System”^27^ for grid calculation and rock mass stress analysis. The model spans a length and width of 4 km, covering an area of 16 km^2^, as shown in Fig. 5.

The stress field of Limin coal mine is characterized by a predominantly horizontal compressive stress, with a maximum compressive stress of 20.6 MPa and azimuth of 265.78°. In the context of rock mass stress calculation and analysis, the maximum principal stress is separately projected onto the *X* and *Y* axes for accurate calculations. The pertinent physical parameters are provided in Table 12.

**Table 12:** Classification and parameters of the top rock lithology of the No. 9 coal seam.

Lithology classification		Elastic modulus/GPa	Poisson ratio
Mudstone		11.04	0.24
Sandy mudstone		19.33	0.22
Siltstone and other lithologies		24.20	0.25
Fault drop height	0–50 m	5.0	0.34
	50–200 m	10.0	0.29

**Figure 6:**
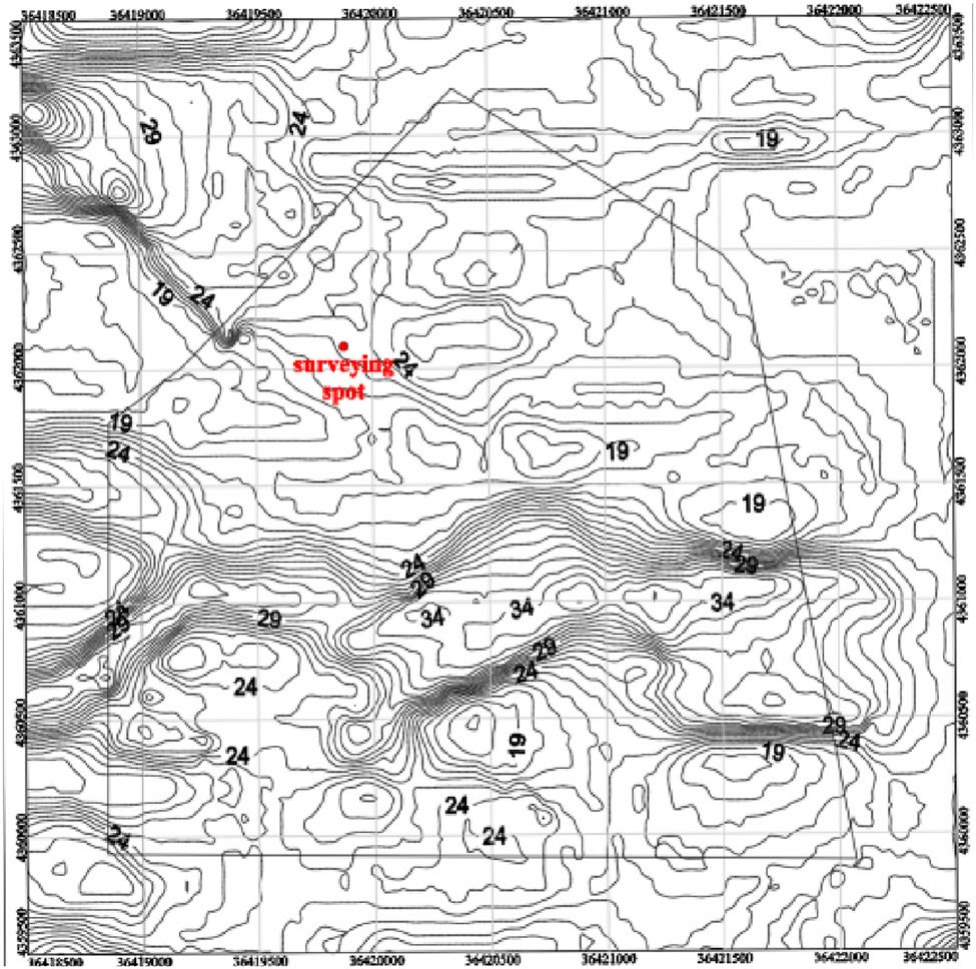
Maximum principal stress isogram of coal seam No. 9 in Limin coal mine.

Using the data mentioned above, we have calculated the rock mass stress of the No. 9 coal seam in Limin coal mine, including the maximum principal stress value, minimum principal stress value, and maximum shear stress value. These results have been exported in a visual format. Additionally, we have generated a visual representation of the maximum principal stress contour map for the No. 9 coal seam in Limin coal mine, as depicted in Fig. 6.

**Figure 7:**
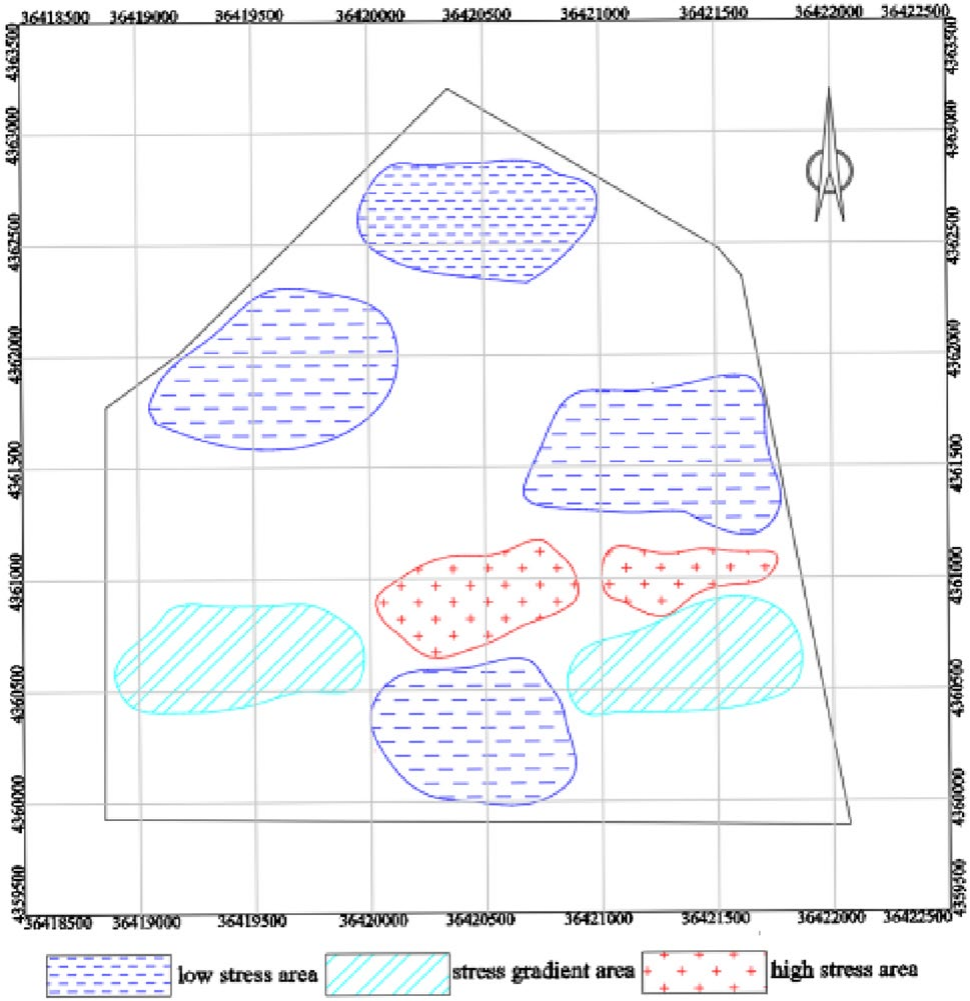
Tectonic stress zoning of the No. 9 coal seam.

The results of the tectonic stress zone delineation, as shown in Fig. 7. Following stress inversion analysis, it was determined that the maximum horizontal principal stress of Coal Seam 9 within the mine field ranges between 14 and 34 MPa. The stress distribution contour line is generated by surfer software, and the rock mass stress zoning is carried out based on the contour line. Zones were categorized based on the stress concentration coefficient (k): high stress zone: k > 1.2, with a maximum stress of 29 to 34 MPa; stress gradient zone: 1 < k ≤ 1.2, with a maximum stress of 24 to 29 MPa; normal stress zone: 0.8 < k ≤ 1, with a maximum stress of 20 to 24 MPa; low stress zone: k ≤ 0.8, with a maximum stress of 14 to 20 MPa.

### The division of tectonic stress danger zone under the synergistic control of fault activation and original rock stress field

Considering the degree of fault activation and the characteristics of original rock stress field, according to the theory of geodynamic zoning, analytic hierarchy process and entropy weight method, the division of tectonic stress danger zone under the synergistic effect of fault activation and original rock stress field in No.9 coal seam of Limin coal mine is comprehensively determined. As shown in Table 13.

**Table 13:** Constructing a comparative matrix for the classification of risk zones.

	Non-activated fault	Critical activated fault	Strongly activated fault	Moderately activated fault	Weakly activated fault
Low stress zone	0	0	1	0	0
Normal stress zone	0	0	1	0	0
Stress gradient zone	1	1	2	1	1
High stress zone	2	2	2	2	2

0-Non-risk zone; 1-Moderate-risk zone; 2-High-risk zone.

**Figure 8:**
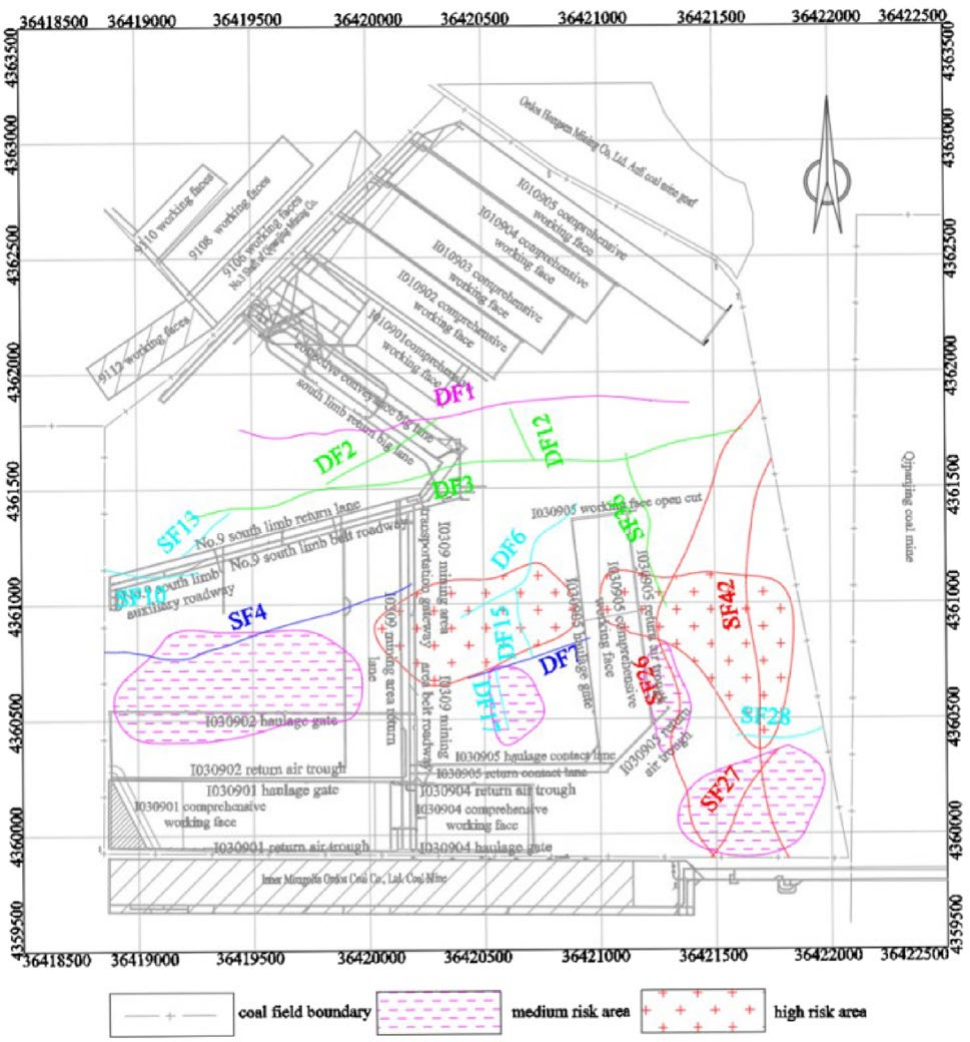
Division of tectonic stress risk zone in No. 9 coal seam of Limin coal mine.

According to the difference of fault activation degree and rock physical and mechanical properties, no, medium and high risk areas are formed in Limin coal mine. The division results of tectonic stress zone under the synergistic effect of fault activation and original rock stress field, as shown in Fig. 8.

1. The high-risk zone predominantly occupies the central and eastern regions of the mine field. The central mine field experiences the impact of critically active fault DF7, resulting in a maximum principal stress ranging from 30 to 34 MPa. Conversely, the eastern mine field is primarily influenced by the original rock stress and the highly active fault SF27, SF42, leading to a maximum principal stress of 29–33 MPa. Consequently, the combined effects of fault activation and original rock stress have significantly augmented the internal energy accumulation compared to unaffected areas, culminating in destabilization and failure of certain coal and rock masses, as well as deformation and damage to the surrounding tunnels. The affected area of this high-risk zone encompasses approximately 14% of the entire mine field’s surface area.
2. The moderate-risk zone is primarily located in the western, southern, and southeastern regions of the mine field. In the western mine field, it is primarily influenced by the critical activity of SF4 fault and its associated original rock stress, resulting in a maximum principal stress range of 24–28 MPa. In the southern part of the mine field, the moderate-risk zone is affected by the strongly active SF26 fault, and the original rock stress, leading to a maximum principal stress value of approximately 24–26 MPa. Similarly, in the southeastern region of the mine field, it is primarily influenced by the strongly active faults SF27 and SF42, resulting in a maximum principal stress range of 20–24 MPa. The combined effects of fault activation and original rock stress have altered the mechanical behavior of the coal and rock masses, leading to a reduction in their limit yield strength. This has made the roadway in these areas more prone to collapse and floor heave. The affected area of this moderate-risk zone constitutes approximately 15% of the total mine field area.
3. The no-risk zone primarily occupies the northern and central regions of the mine field. In the northern part, the zone is influenced by the dual effects of fault activation and original rock stress, resulting in a maximum principal stress range of 14–19 MPa. This combination of factors leads to a decrease in the internal cohesion of the rock, relatively poor energy accumulation, and a shallower mining depth in this area. In contrast, the southern part of the mine field is primarily influenced by the original rock stress, resulting in a maximum principal stress range of 16–20 MPa. The affected area of this no-risk zone constitutes approximately 71% of the total mine field area.

## The influence of roadway stability in different dangerous areas under the coordinated control of fault activation and original rock stress

1. The high-risk area primarily encompasses the central segment of the return air channel of I030905 and the 100 m region west of the transportation channel. Given that the I030905 working face has not yet been mined, the combined effects of original rock stress and mining disturbances are expected to significantly impact the stability of these areas. It is crucial to strengthen the support of the surrounding rock, particularly the floor, to mitigate potential damage and deformation during subsequent excavation and mining operations.
2. The medium-risk zone primarily encompasses most of the I030902 transport tunnel, the northern 150 m of the I030905 transport connection roadway, and the eastern 50 m of the return air roadway. These areas are susceptible to stress disturbances and subsequent tunnel deformation. On-site observations indicate that the I030902 transport tunnel has already experienced floor heave, indicating that the working face is affected by various factors such as mining disturbance. As a result, these areas are prone to roadway damage and instability, requiring additional attention and support measures during excavation operations.
3. The no-danger zone primarily encompasses the entire I0109 mining area, as well as the two roadways within the I030901 and I030904 fully mechanized mining faces, the I030905 face cutting, and most of the transportation troughs. In this region, the stability of the roadways is minimally affected by the original rock stress. However, on-site observations indicate that the deformation and damage of the roadways on both sides of the I030901 face are primarily due to technical factors related to mining. Specifically, the formation of an isolated working face on three sides of this area has resulted in these issues.

## Conclusion

1. Based on the Mohr–Coulomb strength criterion, the fault activation state in No.9 coal seam of Limin Coal Mine is determined, and two states of fault activation are revealed. One is that through activation, the “bonding” state between the upper and lower plates of the fault is changed to the “broken” state; the other is that through activation, the end of the fault and the fault-derived fissures expand, which greatly enhances the permeability of the fault zone and its adjacent rock mass. Thus, the fault activation stress state model is established.
2. Considering the fuzziness of fault research in the theory of geodynamic zoning, the fuzzy comprehensive evaluation method is introduced into the evaluation of fault activation. According to the maximum membership criterion, the membership function of fault activation is determined, and the weight coefficient is determined by the coupling of AHP method and entropy weight method. Finally, the fault activation is comprehensively evaluated.
3. Through in-depth quantitative analysis of the activation characteristics of faults in the mine and rigorous exploration of the distribution patterns of the original rock stress field, the mine field’s tectonic stress danger zone has been carefully classified into three categories: no danger zone, medium danger zone, and high danger zone. These three zones occupy 71%, 15%, and 14% of the mine field area, respectively. This classification is based on a comprehensive assessment of the dual effects of fault activation and original rock stress, providing valuable guidance for safe and efficient mining operations.
4. The interaction between fault activation and original rock stress significantly alters the mechanical behavior of coal and rock masses, leading to dynamic changes in roadway stress distribution and rock strength. This, in turn, compromises the stability of roadways within high- and medium-risk zones. Particularly under conditions of mining disturbance, it is crucial to monitor and support these roadways effectively, with a particular focus on reinforcing the support of the roadway floor. For those roadways that have not yet been put into use, it is essential to take into account the risk zone classification to ensure their tectonic integrity and safety during mining operations.

## Data Availability

All data generated or analysed during this study are included in this published article.
